# Macro- and micro-influencers of antimicrobial costs…What do stewardship programs need to know?

**DOI:** 10.1017/ash.2025.38

**Published:** 2025-02-26

**Authors:** Sheetal Kandiah, Sarah Altamimi, Kristy M Shaeer, Marisa Holubar, Jamie L Wagner

**Affiliations:** 1 Division of Infectious Diseases, Emory University School of Medicine, Atlanta, GA, USA; 2 Grady Health System, Atlanta, GA, USA; 3 Division of Infectious Diseases, Mass General Brigham Salem Hospital, Salem, MA, USA; 4 Department of Pharmacotherapeutics & Clinical Research, University of South Florida Taneja College of Pharmacy, Tampa, FL, USA; 5 Department of Medicine, Division of Infectious Diseases, Stanford Medicine, Stanford, CA, USA; 6 Department of Pharmacy Practice, University of Mississippi School of Pharmacy, Jackson, MS, USA

## Abstract

The high cost of antimicrobials presents critical challenges for healthcare providers managing infections amidst the growing threat of antimicrobial resistance (AMR). High costs hinder access to necessary treatments, disproportionately affecting disadvantaged populations and exacerbating health disparities. High drug prices necessitate the use of less effective or more toxic alternatives, leading to suboptimal outcomes and prolonged hospitalizations. This, in turn, increases healthcare costs and undermines efforts to combat AMR. Equitable policies, national formularies, and cost caps for essential antimicrobials can ensure universal access to life-saving treatments and enable antimicrobial stewardship programs to ensure the best possible outcomes.

## Introduction

Antimicrobial resistance (AMR) adds billions of dollars annually to healthcare expenditures, requiring robust antimicrobial stewardship programs (ASPs) to mitigate these impacts. Between 2000 and 2015, the global use of antimicrobials increased by 65%; primarily due to overconsumption in low- and middle-income countries.^1^ In turn, rising AMR has forced healthcare providers to rely on newer, more expensive antimicrobials.^2^

High costs also lead to delayed or suboptimal treatment.^3^ Rising costs of prescription drugs are deeply felt across the world. Specifically in the United States, 82% of adults believe that medication costs are unreasonable, and 37% of adults report difficulty in affording their prescriptions.^4^ Increasingly common drug shortages further complicate matters, forcing providers to prescribe less effective or more toxic alternatives.^5^ Shortages also contribute to increases in drug pricing, especially when there are limited manufacturers, multiple barriers and few incentives for other companies to manufacture the product, increased demand for the product, and finally, when there is a lack of proven treatment alternatives.^6^ A notable recent example was a national penicillin shortage leading to an abrupt increase in cost amidst a global resurgence of syphilis.^6^

ASPs aim to optimize antimicrobial use and curb resistance while improving quality of care and saving healthcare costs. However, significant disparities in drug access remain due to varying formulary restrictions, insurance coverage, and lack of access to new medications, particularly for marginalized groups already vulnerable to drug-resistant infections.^7^ The complexity of drug pricing, including manufacturer patents and reliance on pharmacy benefit managers, further complicates affordability and access. When developing hospital formularies, drafting guidelines, and building decision support into electronic health records, ASPs should understand the “macro” and “micro” influencers of costs. The purpose of this review is to explore factors determining antimicrobial costs and how financial considerations might influence antimicrobial choices of prescribers. This exploration is crucial for ASPs to understand how best to balance cost-effectiveness with the need to provide life-saving treatments for drug-resistant infections, ultimately ensuring equitable access to effective treatments for all patients.

## “Macro-Influencers” of antimicrobial costs

### Affordable care act

In 2010, approximately 13% of Americans reported unmet prescription drug needs due to cost barriers.^8^ To address this problem, the Patient Protection and Affordable Care Act (ACA) was signed into law in March 2010, creating an individual mandate to have health insurance or face a tax penalty. The ACA was designed to significantly expand Medicaid eligibility and require states to provide Medicaid for the lowest-income U.S. residents. However, in June 2012, the U.S. Supreme Court found that states could not be mandated to participate in the proposed Medicaid expansion, giving states the option either to expand Medicaid under the ACA or to keep their preexisting level of Medicaid benefits without risking loss of federal funding. Unfortunately, this resulted in a “coverage gap” for many uninsured patients who came to rely on safety net healthcare which is often under-resourced or geographically inaccessible.^9^

Prescription drug coverage is an essential benefit under the ACA that does guarantee access to a range of necessary medications. The ACA improved access to 340B drug pricing in Medicaid-expanded states which subsidizes drug pricing for organizations providing care to Medicaid patients. It closed the “donut hole” coverage gaps for patients with Medicare by lowering the overall cost of Medicare Part D prescription plans and required manufacturers to offer 50% rebates on brand name drugs within the coverage gap.

The Affordable Care Act (ACA) also extended federal statutory rebates to prescription drugs provided under Medicaid managed care arrangements, and most states now “carve in” prescription drugs. However, some states have exceptions such as high-cost or specialty drugs.^10^ Further research is necessary to evaluate the impact the ACA has had on antimicrobial prices and access.

### Medicare

Medicare is the federal insurance program in the United States designed for individuals aged 65 and older and some younger individuals with disabilities. While Medicare Parts A and B cover medical services, Part D is a voluntary drug benefit program that enables beneficiaries to obtain prescription medications through private insurance companies. Like commercial insurance, the formulary determines which medications are available to beneficiaries at affordable prices. These formulary decisions are reviewed annually, vary by region, and differ by plan type, affecting all medications, including antimicrobials. This variability can lead to inequitable access and medication costs. For example, those in rural areas tend to have decreased access to life-saving antimicrobials in addition to poor access to clinical trials.^11^ Inadequate access to care prompts clinicians to prescribe unnecessarily broad-spectrum antimicrobials to prevent the need for follow-up visits given geographical limitations.^12^

Cost-sharing tiers classify drugs based on their pricing and management within health plans. Lower-tier drugs are typically less expensive, while higher-tier specialty drugs are significantly costlier. Although many lower-tier antimicrobials are effective for uncomplicated infections, patients with complex medical conditions often require specialty and non-generic antimicrobials falling into a coverage gap, where they must pay a percentage of drug costs after reaching a certain expenditure limit. Individuals on fixed incomes or lower-wage earners are disproportionately impacted by these out-of-pocket expenses. For example, a patient who requires outpatient therapy with an intravenous anti-methicillin-resistant *Staphylococcus aureus* agent and has Medicare PartD coverage might be discharged on daptomycin due to lack of infection resolution with vancomycin. However, since daptomycin is a higher-tier drug than vancomycin, insurance may only cover a certain percentage of this drug (instead of the full cost like with vancomycin).^13,14^ The inability to afford or wait for necessary medications may lead to worsening long-term health outcomes, non-adherence, treatment abandonment and increased hospitalizations, and a higher risk for mortality.^15,16^

Prior authorizations were introduced to help control medical expenses, but it can inadvertently limit or delay access to necessary treatment and lead to adverse outcomes.^17^ In the realm of infectious diseases (ID), timely antimicrobial initiation is crucial for favorable outcomes. Prompt access to antimicrobials can prevent hospitalizations, and timely discharge prescriptions can reduce both length of stay and overall in-hospital costs. However, patients obtaining medications requiring prior authorization can be challenging, heavily relying on medical practice staff to provide justifications promptly, often resulting in delays of up to 72 hours for decisions. This can lead to treatment interruptions or abandonment. For example, dimorphic fungal infections have very specific azoles designated in international guidelines as the drugs of choice; however, many of these “specialty” azoles (eg, posaconazole, itraconazole) are not preferred and/or need trials of ineffective drugs (eg, fluconazole) prior to coverage allowance.^18,19^

Inequities in access to healthcare services and medications are likely to persist as long as disparities among insurance plans remain. A national formulary that is equitable and accessible to all could mitigate these impacts. Just as prescription medications have a cap that can push individuals into the “donut hole”, instituting a cap on out-of-pocket costs for antimicrobial prescriptions could foster greater equity.^20^

It is essential for healthcare providers to remain mindful of costs of antimicrobials and prioritize the most effective and affordable drugs, particularly in under-resourced settings. Providers are often unaware of insurance formulary options. Training programs must include education on formulary restrictions and strategies for selecting effective, affordable treatments. Electronic health records and order entry systems can be programed to include more cost information to guide providers toward optimal choices.

### Medicaid

Medicaid is a joint federal and state program that helps cover medical costs for eligible people with limited income. The federal government has baseline standards for all state Medicaid programs, but each state operates independently which means eligibility and benefits vary between states. In 2010, the ACA expanded Medicaid to nearly all nonelderly adults with income up to 138% federal poverty level ($20,120 annually for an individual in 2023) through a new coverage pathway for adults who had traditionally been excluded from Medicaid coverage. States receive a higher rate of federal funding for people who are enrolled through the new coverage pathway.^21^

Inequities in Medicaid patients are similar to those seen in patients with Medicare part D but are compounded by several additional barriers that create disparities between states. As of June 2024, 41 states have expanded Medicaid but those that have not expanded Medicaid will likely continue to face barriers to non-emergency care with resultant worse health outcomes. This could mean increased hospitalizations due to untreated infections and inability to pay for prescription medications. Quality of care for conditions such as pneumonia is also compromised in safety-net hospitals relative to non-safety-net hospitals.^22,23^

The federal government authorizes states to implement various cost-containment strategies.^24^ For example, a preferred drug list tends to contain lower-cost drugs and those with good rebates. In addition, like Medicare part D, the federal government authorizes states to utilize a prior authorization strategy, however, they must meet two federal criteria: 1) respond to requests for authorization within 24 hours; and, 2) make available a 72-hour supply of medications in an emergency situation. Cost-sharing is another strategy utilized for cost-containment that allows for different pricing for generic or preferred versus non-preferred or brand drugs. However, as previously mentioned, prior authorization requirements place increased burden on healthcare providers, may delay optimal care, and influence provider decision-making in favor of fully covered treatments and diagnostics. For example, bictegravir/emtricitabine/tenofovir alafenamide is a guideline-preferred antiretroviral regimen for patients living with HIV/AIDS (PLWHA). Unfortunately, some state Medicaid programs have this drug listed as a higher tier, or non-preferred, regimen due to its brand-only status.^18,19^

In states with expanded Medicaid coverage, better access to essential antimicrobial treatments may ensue. Urban areas generally have a higher concentration of healthcare facilities, including pharmacies and clinics, which can provide better access to diagnostics and antimicrobials. However, even in urban settings, there may be pockets of underserved communities facing barriers to obtaining affordable treatments due to lower income, pharmacy deserts,^25,26^ and variations in insurance coverage.

To address these challenges, a multifaceted approach is required, involving efforts to enhance provider and pharmacy distribution,^27–30^ such as investing in healthcare infrastructure in rural communities, financially incentivizing healthcare providers to practice in underserved areas, investing in mobile health clinics, implementing telemedicine solutions to bridge the gap, implementing drug regulatory mechanisms to control the unauthorized sale of antimicrobials, and providing financial assistance or subsidies to ensure the affordability of antimicrobials for those in need.^31^

### Commercial health insurance

Commercial health insurance is provided by private issuers and is often employer-sponsored but is available to purchase directly through the marketplace or through professional organizations. Drug prescription costs are a valid concern for policymakers, consumers, and both public and private insurance carriers. In 2017, commercial insurance made up 42% of retail drug spending in the United States. Medicare and Medicaid made up an additional 40% of retail drug spending. In 2016, antimicrobials and asthma/allergy drugs were among the most used drugs under commercial insurance while anti-hypertensive and cholesterol-lowering agents were the most used for Medicare and Medicaid subscribers.^32^

Over the last 10 years, formularies for both government and commercial payors have shrunk. The formularies for commercial payors are likely smaller since they are not held to the same federal mandates that dictate Medicaid and Medicare coverage. Additionally, up to half of medications require a prior authorization ultimately creating delays or abandonment in treatment. Insurance companies will negotiate with manufacturers and make less expensive drugs or those with higher rebates the preferred agents on a formulary. This inherently creates bias and can limit treatment options for consumers who incur out-of-pocket costs. The consumer pays the price through cost-sharing in the form of copays, coinsurance, and deductibles. Kaiser Family Foundation surveyed firms and their largest health plans about cost-sharing tactics; 91% had multiple tiers of prescription coverage while 87% of plans had three or more formulary tiers.^21^ This suggests significant variability in cost-sharing depending on how many and which therapies were indicated. Ultimately, these tactics lead to increased out-of-pocket costs.

### Pharmaceutical companies

A significant challenge to affordable drug costs is the ability of pharmaceutical companies to secure continuation patents on brand-name drugs.^33,34^ This practice delays the introduction of generic drugs, allowing companies to maintain high prices. Further cost analysis is essential to accurately assess the impact of access and cost on consumers in the context of pharmaceutical incentivization. Most believe the increased costs are due to research and development (R&D) interests of the drug companies; however, a recent study demonstrated that R&D investments did not correlate with increased drug prices.^35^

### Pharmacy benefit managers (PBMs)

PBMs are middlemen between pharmaceutical companies, insurers, and pharmacies which act as price drug supply chain negotiators, plan administrators, and key decision-makers who influence which medications will be most accessible to consumers.^20,36^ PBMs create formularies of preferred drugs based on pricing, cost-sharing amounts, and manufacturer rebates that ultimately influence the amount beneficiaries pay out-of-pocket and accessibility of medications on their insurance plans. Both public and private insurers utilize PBM services.^20,36^ Controversy around PBMs and lack of transparency has recently brought PBMs under scrutiny by the Federal Trade Commission alleging that PBMs are profiting off a portion of the rebates rather than passing the full value to the insurer. As a result, independent pharmacies are often unable to survive. This can affect rural populations where pharmacy deserts already exist, further exacerbating the disparate access to medication.

### Summary

Inequities in access to prescription drugs in racial and ethnic minority groups are less likely to receive novel and high-cost medications, lower-cost generic therapies and guideline-established or emergency use treatments, and preventive or critical care therapies.^37,38^ A scoping review of the literature around antimicrobial prescribing revealed similar disparities around antimicrobial prescribing as compared to access to health and diabetes care and pain management.^39,40^ For example, Black patients were less likely than white patients to receive antimicrobials. The variations in prescription drug plans offering various cost-sharing strategies undermine efforts to achieve pharmacoequity for all prescription medications including antimicrobials.^41^ Further research and policy changes are necessary to achieve pharmacoequity which is an integral part of access to healthcare.^26,42^

## “Micro-Influencers” of antimicrobial costs

Healthcare system-level factors, institutional policies, medication formularies, availability of ID specialists, microbiology laboratory support, and availability of social workers and case managers influence outcomes of patients with infectious diseases. Not every institution will have access to ID specialists to select optimal treatments or case managers who can navigate insurance plans and assist with medication access. The electronic order entry system may not be optimized for selection of the shortest effective duration of therapy, and excess antimicrobials may be prescribed at discharge.^43^ Additionally, significant information technology resources and personnel are required to optimize the electronic order entry system, something that not every location has sufficient resources to supply. Electronic order entry system vendors should explore building additional tools and software components that can aid in optimizing antimicrobial prescribing for all institutions, such as adding cost values of antimicrobials to order panels, incorporating patient insurance status into discharge prescription ordering screen, and auto-calculation of antimicrobial durations to incorporate into progress notes.

Starting in 1965, accreditation agencies required hospitals to have a Pharmacy and Therapeutics Committee charged with implementing and managing an inpatient drug formulary system. Whether this committee decides to include a drug on the inpatient formulary depends upon several factors including its therapeutic efficacy, clinical niche, known toxicity, and acquisition cost.^44^ Antimicrobial pricing is complicated and fluid. P&T Committees will evaluate an antimicrobial for formulary status when it is released on the market. While this new antimicrobial is still under patent, its price is relatively stable and set by the manufacturer. After this patent expires, multiple manufacturers will oftentimes produce the drug, driving down its price.^45^ One study found that the price of oral linezolid decreased by 94% after it became generic, and 10 manufacturers began to produce the drug instead of one.^42^ However, the opposite can happen to older, generic drugs that are less frequently used; prices can increase when the number of manufacturers decreases. The same study found most antimicrobials whose price increased by >90% over a 3-year period had ≤ 2 manufacturers.^46^

Drug price can impact cost-effectiveness analyses.^47^ But drug price alone underestimates the true cost of drug utilization, including healthcare provider time, related laboratory costs, and potential costly adverse outcomes. Yoong et al. found that the cost-savings associated with the use of oral ciprofloxacin compared to intravenous ceftriaxone for liver abscesses was driven by reduced clinic visits, not differences in drug prices.^48^ Wagner et al. found that the use of generic daptomycin instead of vancomycin for the same condition saved money and time despite having a higher drug acquisition cost.^49^ This underscores the importance of a dynamic formulary process that is able to reassess the institutional role for drugs when they become generic and their acquisition cost decreases.

Antimicrobial pricing at the local level is complex. Individual healthcare systems or hospitals can negotiate with manufacturers, bundling individual drugs to achieve better pricing or purchasing drugs in bulk. The price of the same drug can also differ by formulation. This means that a healthcare provider who works at several institutions in the same region may have access to different drugs because of each unique formulary. This complexity and lack of transparency creates challenges for individual clinicians who wish to take cost into consideration when making prescribing decisions.^50^

ASPs have long used inpatient formularies to restrict access to certain antimicrobials and have demonstrated associated cost savings.^51^ However, as ASPs focus more and more on generic, relatively low-cost antimicrobials, ASPs must measure their impact in other ways. Studies suggest that as antimicrobials become generic and cheaper, their utilization increases which can impact the emergence of AMR, the healthcare cost of which may be the most expensive of all.^52^

## Prescriber-related factors

**Figure 1.**
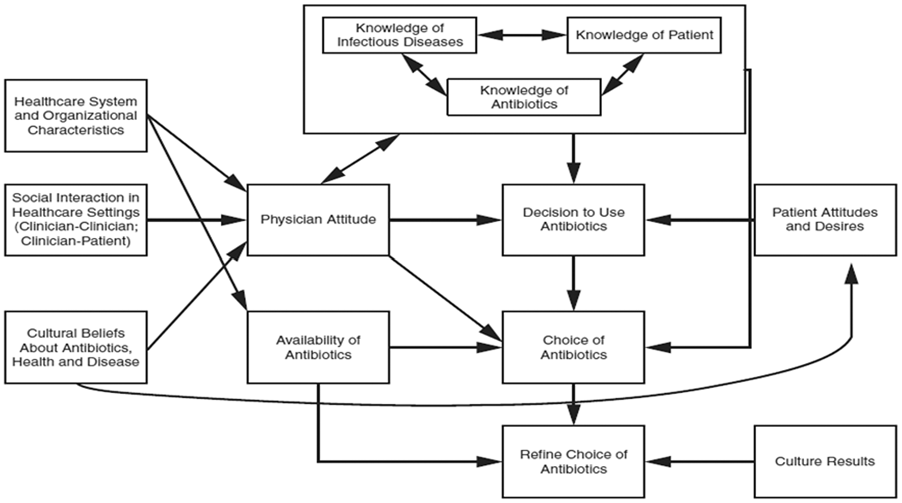
Conceptual framework for antimicrobial use^53^.

Prescribing medications is a complex interaction and clinical decision-making process driven by four key domains: knowledge, resources, social dynamics, and attitudes and perceptions (Figure 1).^53^ These domains may be exacerbated in racial and ethnic minorities, stigmatized populations, and resource-limited settings. For example, differences in antimicrobial prescribing are seen regionally throughout the United States with the highest prescribing rates in the South.^54^ Bizune et al. found that out of 14.9 million clinic visits for acute respiratory tract infections, clinicians prescribed antimicrobials 40% of the time, with higher prescribing in the South (43%) compared to the Midwest (41%), Northeast (37%), and the West (34%).^55^ Qualitative research could provide in-depth insight into the differences in clinical treatment decisions for acute respiratory tract infections. The use of nonprescription antimicrobials may be particularly high among racial and ethnic minorities due to challenges in the accessibility of healthcare services amongst these demographics.^56^ AMR is highly correlated with the use of nonprescription antimicrobials in a community, especially in low- and middle-income countries.^57^ Overprescribing of antimicrobials disproportionally impacts economically disadvantaged countries, communities, and individuals, and overlaps with effects of climate change. Further data are needed to provide more accurate measurements of antimicrobial overuse in low- and middle-income countries, and thus a more accurate measurement of global AMR.

## Conclusion

ASP personnel should be aware of macro- and micro-influencers of antimicrobial costs (Table 1), which in turn, may impact antimicrobial decision-making by prescribers. These factors have substantial implications for patient care. The high cost of newer antimicrobials often limits their use, even when they are necessary to treat resistant infections. Access to high-cost medications is often unequal, with disadvantaged populations facing greater barriers to effective treatment.^58^ The cost of antimicrobials can be a significant barrier to access, particularly for individuals with limited financial resources or inadequate health insurance coverage. This issue is exacerbated by the variations in antimicrobial pricing and reimbursement policies across different regions and states within the United States. Individuals who are unable to afford necessary antimicrobial treatments may be more likely to forgo or delay seeking medical care, potentially leading to the progression of their illness and the development of more severe complications. Addressing these inequities is essential to ensure that all patients benefit from advances in antimicrobial therapy.

**Table 1.** Examples of macro- and micro-influencers on the cost of antimicrobials

	Impact on Antimicrobial Access and Decision-Making
***Macro-influencers on antimicrobial costs***	
Supreme court ruling in 2012 meant that states were no longer mandated to expand Medicaid coverage under the *Patient Protection and Affordable Care Act*, which included prescription drug coverage and access to 340B drug pricing.	“Coverage gap” for prescription drugs in states without Medicaid expansion; Inequitable access and cost to patients
Medicaid, Medicare, and commercial health insurance formularies determine which medications are available to beneficiaries at affordable prices but vary by region and plan type.	Challenging to navigate for prescribers; Inequitable access and cost to patients
Reduction in the number of drugs included on public and private plan formularies and increased number of drugs requiring prior authorizations	Increased burden on prescribers; Limit or delay access to optimal antimicrobials; required trials of less expensive (but more toxic) or ineffective antimicrobials before gold standard therapy is approved
Lack of transparency in practices of Pharmacy Benefit Managers utilized by both public and private insurers	May contribute to independent pharmacy closures, exacerbating pharmacy “deserts”
Pharmaceutical companies’ continuation patents on brand-name antimicrobials	Delay introduction of lower-cost, generic antimicrobials
Antimicrobial shortages	Prescribers forced to use suboptimal antimicrobials
***Micro-influencers on antimicrobial costs (healthcare system levels factors)***	
Local inpatient drug formulary practices vary by institution	Challenging to navigate for prescribers; Inequitable access and cost to patients that may delay discharge
Limited availability of infectious diseases expertise and microbiology laboratory support	Limited familiarity with antimicrobial options
Varied social work and case management support	Limited or delayed access to optimal antimicrobials, especially upon discharge
Lack of transparency of the true cost of an antimicrobial to both institutions and patients when antimicrobials are being prescribed.	Prescribers unable to take cost into consideration during antimicrobial decision-making

We thank members of the SHEA Antimicrobial Stewardship Committee for their review and endorsement of this manuscript.
